# The gut microbiome: A line of defense against tuberculosis development

**DOI:** 10.3389/fcimb.2023.1149679

**Published:** 2023-04-18

**Authors:** Ziqi Yu, Xiang Shen, Aiyao Wang, Chong Hu, Jianyong Chen

**Affiliations:** ^1^ Munich Medical Research School, Ludwig Maximilian University of Munich (LMU), Munich, Germany; ^2^ Department of Gastroenterology and Hepatology, the First Affiliated Hospital of Nanchang Medical College, Jiangxi Provincial People’s Hospital, Nanchang, Jiangxi, China

**Keywords:** gut microbiome, *Mycobacterium tuberculosis*, *Firmicutes*, *Bacteroidetes*, short-chain fatty acids, tuberculosis

## Abstract

The tuberculosis (TB) burden remains a significant global public health concern, especially in less developed countries. While pulmonary tuberculosis (PTB) is the most common form of the disease, extrapulmonary tuberculosis, particularly intestinal TB (ITB), which is mostly secondary to PTB, is also a significant issue. With the development of sequencing technologies, recent studies have investigated the potential role of the gut microbiome in TB development. In this review, we summarized studies investigating the gut microbiome in both PTB and ITB patients (secondary to PTB) compared with healthy controls. Both PTB and ITB patients show reduced gut microbiome diversity characterized by reduced *Firmicutes* and elevated opportunistic pathogens colonization; *Bacteroides* and *Prevotella* were reported with opposite alteration in PTB and ITB patients. The alteration reported in TB patients may lead to a disequilibrium in metabolites such as short-chain fatty acid (SCFA) production, which may recast the lung microbiome and immunity *via* the “gut-lung axis”. These findings may also shed light on the colonization of *Mycobacterium tuberculosis* in the gastrointestinal tract and the development of ITB in PTB patients. The findings highlight the crucial role of the gut microbiome in TB, particularly in ITB development, and suggest that probiotics and postbiotics might be useful supplements in shaping a balanced gut microbiome during TB treatment.

## 1 Introduction

Tuberculosis (TB) caused by *Mycobacterium tuberculosis* is one of the leading infectious disease killers worldwide (Avoi and Liaw, 2021). According to the latest WHO report, it is estimated that a quarter of the global population is infected with *M. tuberculosis*. Even though only about 5-10% of infected people develop active TB, in 2020 alone, the incidence of TB was about 127 cases per 100,000 people, and approximately 1.3 million HIV-negative people died of TB (WHO, 2021). Furthermore, most TB cases were reported in less developed regions, especially in South-East Asia, Africa, and the Western Pacific regions (WHO, 2021). However, the incidence might be underestimated as in some areas, especially in sub-Saharan Africa, the diagnosis of TB is still a challenge, and it is estimated that approximately 50% of TB cases remain undiagnosed (Mnyambwa et al., 2021; Jayasooriya et al., 2022). In the year 2015, all WHO members adopted the WHO’s End TB strategy which aims to reduce the absolute number of TB deaths by 95% and the incidence rate by 90% by 2035 compared to the 2015 baseline. Six years have passed, and the incidence of TB has only dropped by 10%. With only 13 years left, the situation is still challenging.

TB is transmitted by cough-generated aerosols from patients, and it primarily affects the lungs, causing pulmonary tuberculosis (PTB) (Tan et al., 2020). However, it can also involve other parts of the body. TB that affects areas outside the lungs is called extrapulmonary tuberculosis. Approximately 1-3% of total TB cases (Sheer and Coyle, 2003; Cho et al., 2018) and 10% of all extrapulmonary tuberculosis cases involve the gastrointestinal tract, causing intestinal tuberculosis (ITB) (Abu-Zidan and Sheek-Hussein, 2019; Maulahela et al., 2022). Swallowing of sputum in PTB patients has a certain chance of causing ITB (Gan et al., 2016). This is because *M. tuberculosis* is more resistant to the gastric acid barrier due to its special cell wall structure (Vandal et al., 2009). However, not all PTB patients develop ITB, as they might benefit from the protective effect of the intestinal barrier.

The intestinal barrier is a highly complex system, including the outer mucus layer, the epithelial layer, the underlying lamina propria, and components such as commensal microbiota, antimicrobial peptides, secretory immunoglobulin A, and immune cells (König et al., 2016; Vancamelbeke and Vermeire, 2017). Intestinal microbiota with a complex and dynamic microbial community is of vital importance to human health (Chen et al., 2021). It can not only regulate host physiological processes such as digestion, nutrient absorption, and metabolism, but also modulate host immunity in protection against pathogens and toxins (Wang et al., 2017; Comberiati et al., 2021). It is of great importance in gut homeostasis and colonization resistance to exogenous pathogens (Ducarmon et al., 2019), and dysbiosis in microbiome composition can result in susceptibility to infections and disease development (Budden et al., 2017). It is reported that altered microbiota composition can cause increased epithelial permeability and disruption in the mucus layer, resulting in susceptibility to *Clostridioides difficile* (Bien et al., 2013) and *Citrobacter rodentium* infection (Wlodarska et al., 2011). A recent study in patients with COVID-19 observed significant gut dysbiosis with enrichment of opportunistic pathogens (Zuo et al., 2020). Therefore, the gut microbiome of the host might also be crucial in preventing TB infection or decelerating the disease progression (Hu et al., 2019b).

With the universal application of Next-Generation Sequencing and bioinformatic analysis, there are increasing studies investigating the association between *M. tuberculosis* infection and alteration of gut microbiota. Here, we reviewed all the previous reports on the intestinal microbiome in active TB patients (including PTB and ITB) without any treatment, summarized their main findings, and tried to deduce the reasons for ITB development in PTB patients.

## 2 Alteration of gut microbiome in active TB patients

**Figure 1:**
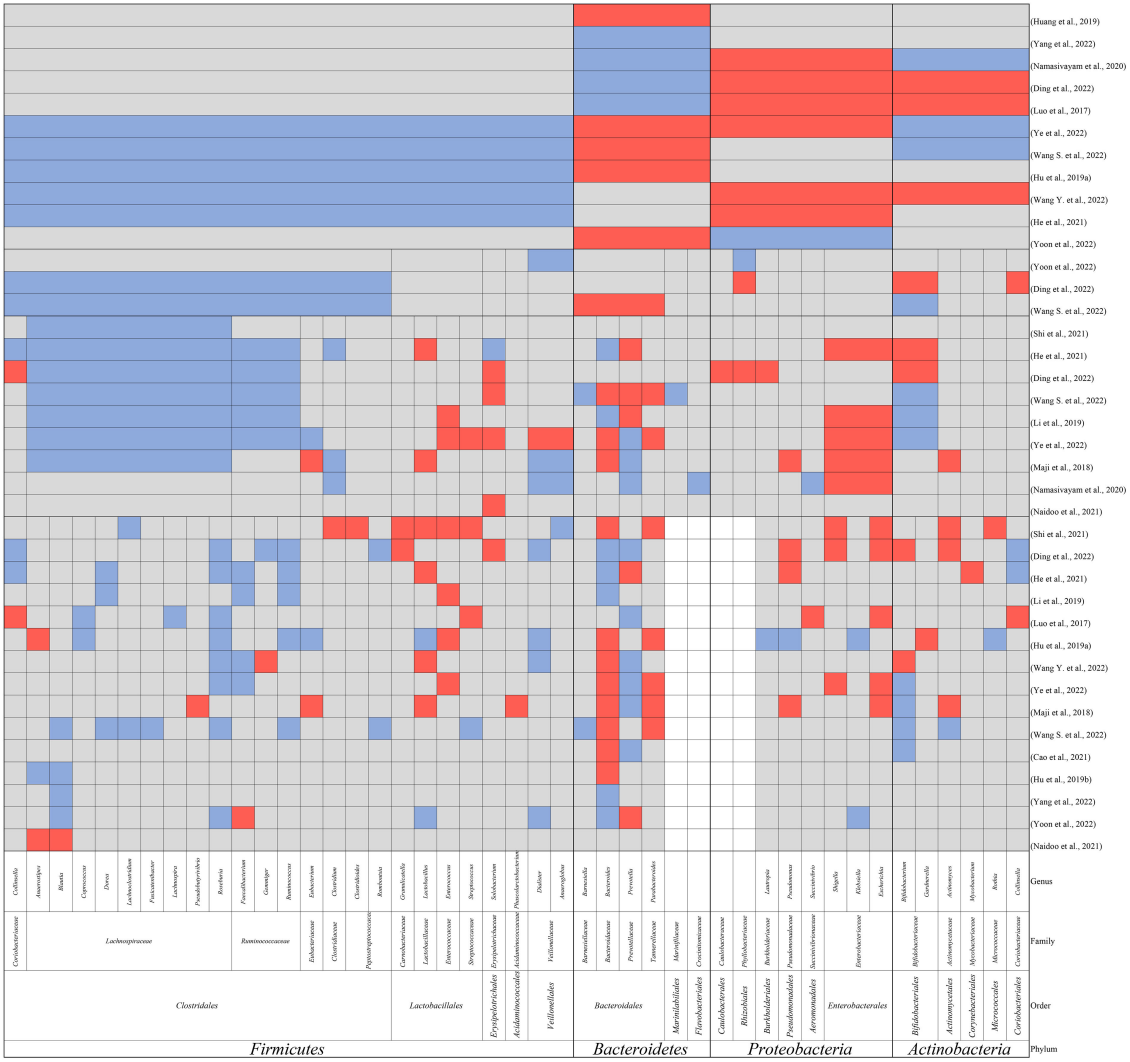
The main findings in alteration of gut microbiomes in TB patients compared to healthy controls at the phylum, order, family, and genus level. Red: elevation; blue: reduction; grey: not reported; white: no reported genus within the family.

*M. tuberculosis* infection is known to cause dysregulation of the immune system, resulting in dysregulation of the gut microbiome (Osei Sekyere et al., 2020). In this review, we included studies referring to the alterations in the gut microbiome of TB patients (Luo et al., 2017; Maji et al., 2018; Huang et al., 2019; Hu et al., 2019a; Hu et al., 2019b; Li et al., 2019; Namasivayam et al., 2020; Cao et al., 2021; He et al., 2021; Naidoo et al., 2021; Shi et al., 2021; Ding et al., 2022; Wang S. et al., 2022; Wang Y. et al., 2022; Yang et al., 2022; Ye et al., 2022; Yoon et al., 2022). All patients included in the study were without antibiotic treatment, as the antibiotics can result in dysbiosis and mask the results caused by *M. tuberculosis* infection (Hu et al., 2019a; Namasivayam et al., 2020). The main findings are summarized in **Table 1** and **Figure 1**. The study design and sequencing techniques used in these studies are also included.

**Table 1:** Studies investigating the alteration of gut microbiome in pulmonary tuberculosis patients or intestinal tuberculosis patients without antibiotics comparing with the healthy controls.

Study design		Change in diversity	Change in microbiota composition	Sequencing technology	Literature
Patients	Controls				
Stool samples from active PTB patients (n=29)	Stool samples from healthy controls (n=22)	decreased alpha-deversity	*Bifidobacterium* and *Prevotella* decreased in patients	16S rRNA gene amplicon (Illumina) sequencing	(Cao et al., 2021)
			*Bacteroidetes* increased in patients		
Stool samples from PTB patients (n=10)	Stool samples from healthy controls (n=20)	decreased alpha-deversity	*Bacteroidetes*, *Clostridales*, *Ruminococcaceae*, *Lachnospiraceae*, *Prevotella*, *Romboutsia*, *Dialister*, *Gemmiger*, *Collinsella* and *Roseburia* decreased in patients;	16S rRNA gene amplicon (Illumina) sequencing	(Ding et al., 2022)
			*Proteobacteria*, *Actinobacteria*, *Bifidobacteriales*, *Coriobacteriales*, *Rhizobiales*, *Bifidobacteriaceae*, *Coriobacteriaceae*, *Caulobacteraceae*, *Phyllobacteriaceae*, *Burkholderiaceae*, *Granulicatella*, *Solobacterium*, *Erysipelotrichaceae unclassified* and *Actinomyces* increased in patients		
Colon biopsy samples from ITB patients (n=6)	Colon biopsy samples from healthy controls (n=4)	no significant difference	*Firmicutes*, *Lachnospiraceae*, *Ruminococcaceae*, *Bacteroidaceae*, *Bacteroides*, *Faecalibacterium*, *Roseburia*, *Collinsella*, *Dorea*, *Oscillibacter*, *Ruminococcus* decreased in patients;	16S rRNA gene amplicon (Illumina) sequencing	(He et al., 2021)
			*Proteobacteria*, *Enterobacteriaceae*, *Lactobacillus*, *Pseudomonas*, *Klebsiella*, *Mycobacterium* increased *in patients*		
Stool samples from PTB patients (n=30)	Stool samples from healthy controls (n=52)	decreased alpha-deversity	*Roseburia hominis*, *Roseburia inulinivorans*, *Roseburia intestinalis*, *Eubacterium rectale*, *Coprococcus comes*, *Bifidobacterium adolescentis*, *Bifidobacterium longum*, *Ruminococcus obeum*, *Akkermansia muciniphila*, *Haemophilus parainfluenzae* decreased in patients;	Shotgun metagenomic Illumina sequencing	(Hu et al., 2019a)
			unclassified *Coprobacillus bacterium*, *Clostridium bolteae* increased in patients		
Stool samples from active PTB patients (n=28), latent PTB (n=10)	Stool samples from healthy controls (n=13)	minor decreased alpha-deversity	*Bacteroides* slightly increased in patients	16S rRNA gene amplicon (Illumina) sequencing	(Hu et al., 2019b)
Stool samples from active PTB patients (n=25), latent PTB (n=32)	Stool samples from healthy controls (n=23)	not reported	*Firmicutes*/*Bacteroidetes* ratio decreased in patients;	16S rRNA gene amplicon (Illumina) sequencing	(Huang et al., 2019)
			*Bacteroidetes* increased in patients		
Stool samples from PTB patients (n=18)	Stool samples from healthy controls (n=18)	decreased alpha-deversity	*Bifidobacteriaceae*, *Ruminococcaceae*, *Bacteroidaceae*, *Faecalibacterium*, *Faecalibacterium prausnitzii* decreased in patients;	16S rRNA gene amplicon (454) pyrosequencing	(Li et al., 2019)
			*Prevotellaceae*, *Enterococcus* increased in patients		
Stool samples from new PTB patients (n=19), recurrent PTB (n=18)	Stool samples from healthy controls (n=20) but with younger age structure and more female	increased alpha-diversity	*Bacteroidetes* and *Coprococcus* depletion in RTB and NTB;	16S rRNA gene amplicon (Illumina) sequencing	(Luo et al., 2017)
			*Firmicutes* decreased in RTB, *Roseburia* decreased in NTB, *Lachnospira* and *Prevotella* decreased in both NTB and RTB patients;		
			*Actinobacteria*, *Proteobacteria*, *Streptococcus* increased in both NTB and RTB patients, *Escherchia* and *Collinsella* increased in RTB		
Stool samples from PTB patients (n=6)	Stool samples from healthy blood relatives of each patient (n=6)	decreased alpha-deversity	*Bifidobacterium* decreased and *Prevotella* depletion in patients;	16S rRNA gene amplicon (Illumina) sequencing; faecal whole genome shotgun sequencing (Illumina)	(Maji et al., 2018)
			*Faecalibacterium*, *Coprococcus*, *Phascolarctobacterium*, *Pseudobutyrivibrio*, *Bacteroides*, *Eubacterium rectale*, *Phascolarctobacterium succinatutens*, *Roseburia inulinivorans*, *Faecalibacterium prausnitzii*, *Shigella sonnei*, *Escherichia Coli*, *Streptococcus pneumoniae*, *Streptococcus vestibularis* were increased in patients		
Stool samples from PTB patients (n=58) and symptomatic controls (n=47)	Stool samples from close contacts PTB cases (n=73) and close contacts of symptomatic controls (n=82)	inconclusive	*Erysipelotrichaceae*, *Anaerostipes* and *Blautia* increased in patients	16S rRNA gene amplicon (Illumina) sequencing	(Naidoo et al., 2021)
Stool samples from new *M. tuberculosis* PTB patients (n=21)	Stool samples from healthy controls (n=10)	decreased alpha-deversity	*Bacteroidetes*, *Actinobacteria*, *Veillonellaceae*, *Succinivibrionaceae* and *Crocinitomicaceae* decreased in patients	16S rRNA gene amplicon (Illumina) sequencing	(Namasivayam et al., 2020)
Stool samples from PTB patients with antibiotics (n=39) and PTB patients without antibiotics (n=55)	Stool samples from TB negative controls (n=62)	decreased alpha-deversity	*Lachnospiraceae*, *Lachnoclostridium*, *Anaeroglobus* decreased in PTB patients without antibiotics;	16S rRNA gene amplicon (454) pyrosequencing	(Shi et al., 2021)
			*Enterococcus*, *Clostridiales* and *Rothia* increased in patients		
Stool samples from new PTB patients (n=83)	Stool samples from healthy controls (n=31)	decreased alpha-deversity	*Firmicutes*, *Actinobacteria*, *Clostridales*, *Bifidobacteriales*, *Bifidobacteriaceae*, *Lachnospiraceae*, *Ruminococcaceae*, *Marinifilaceae*, *Eggerhellaceae*, *Barnesiellaceae*, *Blautia*, *Roseburia, Bifidobacterium*, undifined *Ruminococcaceae*, *Fusicatenibacter*, *Romboutsia* decreased in patients;	16S rRNA gene amplicon (454) pyrosequencing	(Wang S. et al., 2022)
			*Bacteroidetes*, *Bacteroidales*, *Bacteroidaceae*, *Tannerellaceae*, *Fusobacteriaceae*, *Erysipelotrichaceae*, *Prevotellaceae*, *Bacteroides*, *Parabacteroides*, *Fusobacterium*, *Lachnoclostridium*, *Bacteroides vulgatus* increased in patients		
Stool samples from new PTB patients (n=56) and latent PTB (n=36)	Stool samples from healthy controls (n=50)	decreased alpha-deversity	*Firmicutes*, *Tenericutes*, *Roseburia* decreased in patients;	16S rRNA gene amplicon (Illumina) sequencing	(Wang Y. et al., 2022)
			*Actinobacteria*, *Bifidobacterium* increased in patients		
Stool samples from new PTB patients (n=55)	Stool samples from healthy controls (n=50) with slightly younger median age	decreased alpha-deversity	*Bacteroidetes* and *Bacteroides fragilis* decreased in patients	RT-qPCR for targeting certain phylum, family or species	(Yang et al., 2022)
Stool samples from PTB patients (n=69)	Stool samples from healthy controls (n=10)	decreased alpha-deversity	*Bacteroidetes, Proteobacteria, Fusobacteria, Bacteroidaceae, Tannerllaceae, Bacteroides, Veillonella* increased in patients	16S rRNA gene amplicon (515, 806) pyrosequencing	(Ye et al., 2022)
			*Firmicutes, Actinobacteria, Bifidobacteriaceae, Butyricioccaceae, Ruminococcaceae, Faecalibacterium, Bifidobacterium, Agathobacter* decreased in patients		
Stool samples from ITB patients (n=11)	Stool samples from healthy controls (n=63)	decreased alpha-deversity	*Proteobacteria*, *Megasphaera*, *Veillonellales* decreased in patients	16S rRNA gene amplicon (Illumina) sequencing	(Yoon et al., 2022)
			*Verrucomicrobia*, *Rhizobiales*, *Blautia* increased in patients		

Most of the studies found a decreased alpha-diversity in TB patients (Maji et al., 2018; Hu et al., 2019a; Hu et al., 2019b; Li et al., 2019; Namasivayam et al., 2020; Cao et al., 2021; He et al., 2021; Shi et al., 2021; Ding et al., 2022; Wang S. et al., 2022; Wang Y. et al., 2022; Yang et al., 2022; Ye et al., 2022; Yoon et al., 2022), with only one exception reporting increased diversity in both newly diagnosed PTB and recurrent PTB patients (Luo et al., 2017). However, it should be noted that the study by Luo et al. reported a significant difference in the age structure between the healthy control group and the two TB patient groups (Luo et al., 2017), which might have contributed to the observed enhancement in gut microbiome diversity. In a mouse model challenged with *M. tuberculosis*, dysbiosis resembling that observed in TB patients was observed in TB patients was reported (Winglee et al., 2014). The authors found a rapid initial post-infection reduction in alpha-diversity of the gut microbiome followed by slight recovery of diversity until death (Winglee et al., 2014). They proposed that the change in gut microbiome was due to the crosstalk between microbiota and immune system activation, while the recovery of diversity indicated the attainment of balance.

The dysbiosis observed in the gut microbiome of TB patients at the taxonomic level was mainly in the following aspects.

### 2.1 Firmicutes

*Firmicutes*, which play a role in nutrition and metabolism (Stojanov et al., 2020), are the most abundant microbiome in the healthy human colon, comprising 64% of the gut microbiome (Piccioni et al., 2022). The imbalance in the ratio of *Firmicutes/Bacteroides* was also reported to indicate disrupted intestinal homeostasis, pathogen invasion, or unhealthy conditions (Stojanov et al., 2020). The significant reduction in the phylum *Firmicutes* in TB patients was observed by several independent groups (Hu et al., 2019a; He et al., 2021; Wang S. et al., 2022; Wang Y. et al., 2022; Ye et al., 2022). The relationship between reduced *Firmicutes* and *M. tuberculosis* infection might be regarded as reciprocal causation. On one hand, the imbalanced microbiome composition caused by *Firmicutes* reduction might cause susceptibility to *M. tuberculosis* infection or the activation of TB in latent TB infection. On the other hand, the reduction of *Firmicutes* might also be triggered by the dysregulated immune system caused by *M. tuberculosis* infection.

Precisely, within *Firmicutes*, *Clostridiales* and *Veillonellales* were found to be decreased by some studies (Ding et al., 2022; Wang S. et al., 2022; Yoon et al., 2022). Meanwhile, many observations support the reduction of families *Lachnospiraceae*, *Ruminococcaceae*, and *Clostridiaceae* within *Clostridiales* (Maji et al., 2018; Li et al., 2019; He et al., 2021; Shi et al., 2021; Ding et al., 2022; Wang S. et al., 2022; Ye et al., 2022) and the reduction of *Veillonellaceae* within *Veillonellales* (Maji et al., 2018; Namasivayam et al., 2020). More interesting findings were observed at the genus level. Some of the most common genera in *Firmicutes* such as *Faecalibacterium*, *Ruminococcus*, *Blautia*, *Roseburia*, *Lachnospira*, *Eubacterium*, *Coprococcus*, and *Dorea* were all observed to be decreased (Luo et al., 2017; Hu et al., 2019a; Hu et al., 2019b; Li et al., 2019; He et al., 2021; Shi et al., 2021; Ding et al., 2022; Wang S. et al., 2022; Wang Y. et al., 2022; Yang et al., 2022; Ye et al., 2022; Yoon et al., 2022), whereas *Granulicatella*, *Lactobacillus*, *Enterococcus*, and *Streptococcus* were observed to be increased in patients (Luo et al., 2017; Maji et al., 2018; Hu et al., 2019a; Li et al., 2019; He et al., 2021; Shi et al., 2021; Ding et al., 2022; Wang Y. et al., 2022; Ye et al., 2022).

As mentioned earlier, the reduced genera primarily belong to the two most abundant families in *Firmicutes*, *Lachnospiraceae* and *Ruminococcacea*e. They are obligate anaerobic and butyrate-producing bacteria (Sorbara et al., 2020; Liu et al., 2021). Butyrate is a short-chain fatty acid (SCFA) that is an essential regulator for the maintenance of intestinal homeostasis (Parada Venegas et al., 2019). Butyrate can interact with G-coupled receptors such as GPR43, GPR41, and GPR109a (Hodgkinson et al., 2023), leading to increased regulatory T cells (Tregs) and dendritic cell precursors, improved epithelial barrier function, as well as the increased expression of anti-inflammatory cytokines such as IL-10 (Liu et al., 2018). Additionally, butyrate can also inhibit HDAC activity to decompact chromatin and upregulate gene expression, inducing Tregs and the antimicrobial activity in intestinal macrophages (Schulthess et al., 2019). In addition, Phenylbutyrate (PBA), a derivative of butyrate, has been found to induce the expression of antimicrobial peptides in lung epithelial cells (Steinmann et al., 2009) and directly restrict the growth of *M. tuberculosis in vitro* or even within macrophages (Coussens et al., 2015). In clinical trials for TB patients, PBA in combination with vitamin D has also been shown to increase the clearance of *M. tuberculosis* by inducing the antimicrobial peptide LL-37 (Mily et al., 2013; Mily et al., 2015), while also ameliorating inflammation and improving symptom relief (Bekele et al., 2018; Rekha et al., 2018). LL-37 was reported to disrupt the cell wall of intra- and extracellular *M. tuberculosis* (Deshpande et al., 2020) and also activate the autophagy of macrophages (Rekha et al., 2015). Therefore, a decreased butyrate level would result in elevated pro-inflammatory responses, reduced antimicrobial activity, and impaired epithelial barrier function (Chen et al., 2019b).

Conversely, the increased genera in patients all belong to the order *Lactobacillales*, a group of lactic acid-producing bacteria. Lactic acid bacteria are generally regarded as beneficial microorganisms that support the host’s gut homeostasis and enhance the epithelial barrier (Ren et al., 2020). However, it is also reported that lactic acid bacteria can induce Th1 and suppress Th2 responses during *M. tuberculosis* infection (Ghadimi et al., 2010). Meanwhile, it is also worth noting that some of the bacteria in *Enterococcus*, *Streptococcus*, and *Granulicatella* are opportunistic pathogens. The disrupted epithelial barrier caused by reduced butyrate can facilitate the colonization of these opportunistic pathogens.

### 2.2 Bacteroidetes

*Bacteroidetes* are the second most abundant microbiota in the healthy human colon, comprising 23% of the gut microbiota (Sánchez-Tapia et al., 2019). Similar to *Firmicutes*, alterations in *Bacteroidetes* are also important in metabolism and energy balance (Chen et al., 2019a). However, unlike *Firmicutes*, *Bacteroidetes* are the main producer of the other two members of SCFAs, namely acetate and propionate (Feng et al., 2018).

Despite the contradictory findings in the alteration of *Bacteroidetes*, the most predominant findings were related to the three most abundant genera in *Bacteroidetes*, namely *Bacteroides*, *Prevotella*, and *Parabacteroides* (Rinninella et al., 2019; Zafar and Saier, 2021). In most studies, *Bacteroides* and *Parabacteroides* were reported to be increased in TB patients while *Prevotella* was reported to be decreased (Maji et al., 2018; Hu et al., 2019a; Hu et al., 2019b; Shi et al., 2021; Wang S. et al., 2022; Wang Y. et al., 2022; Ye et al., 2022).

Both *Bacteroides* and *Parabacteroides* are acetate-producing bacteria. Like butyrate, acetate can enhance antimicrobial peptides such as defensins, and also increase the epithelial barrier repairment by inducing the production of IL-22 (Fachi et al., 2020). Defensin, such as defensin-1, was found to inhibit the intracellular growth of mycobacterium inside granulomas (Sharma et al., 2017). Moreover, acetate was also reported to increase phagocytosis and bacterial killing by macrophages and neutrophils (Galvão et al., 2018). In addition, *Bacteroides* was also one of the major sources of propionate in the gut microbiota (Louis and Flint, 2017). Propionate was also shown to have antimicrobial activity. Propionate produced by *Bacteroides* was reported to limit the colonization of many bacteria such as *Salmonella* (Jacobson et al., 2018) and *E.coli* (Ormsby et al., 2020) by regulating intracellular pH. However, it should not be neglected that acetate may also suppress CD4+ T cell activation and Th1 and Th17 response while propionate may suppress antigen-specific CD8+ T cell activation by alleviating the IL-12 production by dendritic cells (Nastasi et al., 2017). These effects may also increase the susceptibility of the host to infections (Ahn et al., 2017; Piccinni et al., 2019).

In contrast, studies have shown that *Prevotella* can augment Th17-mediated mucosal inflammation (Kempski et al., 2017) and increase epithelial permeability to bacterial products (Larsen, 2017). This might be because *Prevotella* can activate TLR2-signaling and induce the secretion of IL-6, IL-8, and CCL20 by epithelial cells (Tamanai-Shacoori et al., 2022), as well as the secretion of IL-1β, IL-6, and IL-23 by dendritic cells (Kwok et al., 2012). These cytokines can induce Th17 immune response and neutrophil recruitment, increasing infection severity and tissue damage (Larsen, 2017; Shen and Chen, 2018). Therefore, reduced *Prevotella* as well as increased *Bacteroides* and *Parabacteroides* might simultaneously exert an anti-inflammatory effect.

Intriguingly, in the context of ITB, there seems to be minor differences compared with PTB patients. The most significant observation would be the opposite trends with decreased *Bacteroides* and increased *Prevotella* in ITB patients (He et al., 2021; Yoon et al., 2022). As the major sources of both acetate and propionate, decreased *Bacteroides* together with downregulated *Firmicutes* in ITB patients would result in a dramatic depletion of SCFA production. Based on the critical role that SCFAs play in epithelial barrier function, antimicrobial protein production, and immunomodulation, this depletion would cause excessive immune responses, increased inflammatory lesions, and antimicrobial peptide production. It might also increase the invasion and colonization of *M. tuberculosis* and other opportunistic pathogens in the gut.

Moreover, the increased *Prevotella* would also increase the Th17 response inducing neutrophil accumulation and granuloma formation after *M. tuberculosis* infection (Seiler et al., 2003). However, when exposed to excessive IL-17 produced by Th17 cells, longer survival of neutrophils can cause increased neutrophil infiltration and the formation of pathological lesions (Torrado and Cooper, 2010). This is also in line with the observation of elevated IL-17 expression in ITB patients (Pugazhendhi et al., 2013).

### 2.3 *Proteobacteria* and *Actinobacteria*

At the phylum level, *Proteobacteria* were observed to be increased in TB patients (Luo et al., 2017; Namasivayam et al., 2020; He et al., 2021; Ding et al., 2022; Wang Y. et al., 2022), while conflicting trends were reported for *Actinobacteria* (Luo et al., 2017; Namasivayam et al., 2020; Ding et al., 2022; Wang S. et al., 2022; Wang Y. et al., 2022). However, at the genus level, *Pseudomonas (* Maji et al., 2018; He et al., 2021; Shi et al., 2021 *)*, *Shigella* (Shi et al., 2021; Ding et al., 2022)and *Escherichia* from *Proteobacteria* (Luo et al., 2017; Shi et al., 2021; Ding et al., 2022)and *Actinomyces* from *Actinobacteria* (Maji et al., 2018; Shi et al., 2021; Ding et al., 2022)were all reported to be increased in patients. These bacteria are all common opportunistic pathogens and are always associated with the disruption of mucosal barriers (Pujic et al., 2015). An imbalanced SCFA constitution alters the gut environment resulting in dysregulated immune response and breakdown of the epithelial barrier, causing the colonization of opportunistic pathogens.

## 3 Microbiome-immune crosstalk during *M. tuberculosis* infection

The gut microbiome and lung microbiome are not separate groups within an organism. They are tightly related by the so-called “gut-lung axis”, which means that the metabolites produced by the gut microbiome can reach the systemic circulation and shape the lung microbiome and the immune response in the lung, and vice versa (Enaud et al., 2020). Among the metabolites of the microbiome, SCFAs are the most extensively studied. SCFAs including acetate, propionate, and butyrate have been shown to have a modulatory role in the immune system and epithelial function.

**Figure 2:**
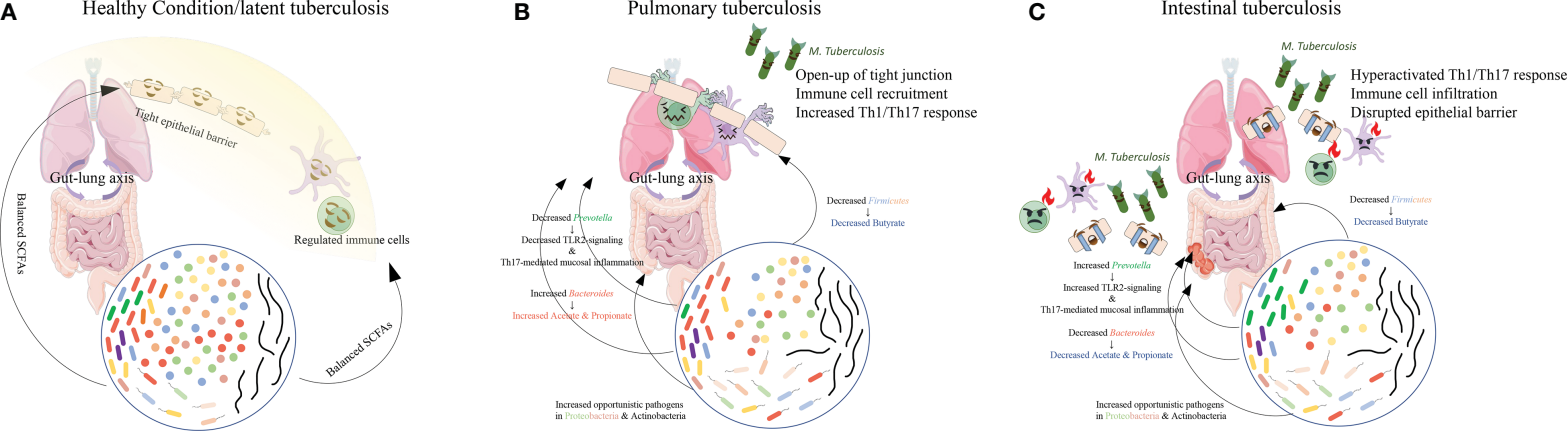
The main findings in gut microbiome composition in PTB and ITB patients. Compared with the healthy conditions **(A)**, in PTB patients **(B)**, reduced *Firmicutes* and *Prevotella* and increased *Bacteroides* altered the proportion of each SCFA, causing immune cell recruitment and mildly increased immune response. However, when *Bacteroides* decreased and *Prevotella* increased **(C)**, decreased SCFAs production resulted in drastic activation of immune response and disruption of epithelial barrier, facilitating the colonization of *M. tuberculosis* in the intestine and the development of ITB.

In PTB patients, compared with healthy controls (**Figure 2A**), the main findings are the loss of *Firmicutes* such as *Lachnospiraceae* and *Ruminococcaceae*, and the enrichment of *Bacteroidetes* (**Figure 2B**). In the murine model challenged with *M. tuberculosis*, the authors also observed a post-infection reduction of butyrate-producing *Lachnospiraceae* and *Ruminococcaceae* and enrichment of acetate/propionate-producing *Bacteroides*, similar to the observations in humans (Winglee et al., 2014). Furthermore, two studies on the relationship between *Helicobacter hepaticus* and *M. tuberculosis infection* found that infection by *Helicobacter hepaticus* resulting in similar dysbiosis with increased *Bacteroidaceae* and decreased *Clostridiales*, *Ruminococcaceae*, *Lachnospiraceae*, and *Prevotellaceae* could cause hyperactivated immune response, overexpressed pro-inflammatory cytokines, and increased susceptibility to *M. tuberculosis*, resulting in severe lung damage (Arnold et al., 2015; Majlessi et al., 2017). These observations in patients and murine models may lead to the potential altered SCFA composition with decreased butyrate but increased acetate and propionate. A fecal metabolomic study also revealed slightly increased acetate and a significant decrease in butyrate in PTB patients (Wang S. et al., 2022).

Acetate, butyrate, and propionate are all SCFAs that can exert anti-inflammatory effects by binding to GPR41 and GPR43. However, butyrate is the only SCFA known to bind to GPR109A (Liu et al., 2018). *In vivo* experiments using *Gpr109a* ^-/-^ mice failed to ameliorate the inflammatory response and epithelial barrier dysfunction after sodium butyrate administration (Chen et al., 2018), indicating the importance of GPR109A in anti-inflammatory response and epithelial barrier construction. Another experiment using *Gpr109a* ^-/-^ mice observed dysregulated immune responses and increased M1 macrophage polarization (Zhang Z. et al., 2022). Increased acetate and propionate may remedy the loss of butyrate in GPR41 and GPR43 activation but may not rescue the loss of GPR109A activation. The loss of butyrate in the gut microbiome and further in the circulation by the “gut-lung axis” results in dysbiosis in the lung microbiome (Hu et al., 2020; Vázquez-Pérez et al., 2020; Xiao et al., 2022; Zhang M. et al., 2022), as well as the disruption of the lung epithelial barrier and upregulation of pro-inflammatory cytokines in the systemic circulation such as IFN-γ, TNF, and IL-17A (Machado et al., 2021). These pro-inflammatory cytokines and the opening up of tight junctions in the lung epithelial barrier can facilitate the migration of immune cells such as neutrophils and macrophages (Akdis, 2021). Macrophages and neutrophils are the first-line innate immune defense against *M. tuberculosis* by phagocytosis (Roca et al., 2019). Moreover, immune cells such as macrophages and dendritic cells can present antigens to T and B cells and augment adaptive immune responses. After infection, CD4+ T cells can not only further strengthen the innate immunity but also promote the function and survival of CD8+ T cells (Lu et al., 2021), whilst CD8+ T cells can directly kill *M. tuberculosis* by their cytolytic function (Lin and Flynn, 2015). Antibody opsonization was also shown to promote the phagocytosis of macrophages (Chandra et al., 2022).

However, when the SCFA level in circulation is sustainably reduced due to an imbalanced microbiome in TB, as observed in ITB patients with decreased Bacteroides (**Figure 2C**), the resulting depletion of IL-10 production and anti-inflammatory response can provoke the persistence of an overactivated pro-inflammatory response. Meanwhile, excessive TNF production was found to induce necroptosis of granuloma macrophages by activating the RIP1-RIP3 necroptosome (Stutz et al., 2018), which can facilitate bacterial replication and activation (Roca et al., 2019). Moreover, the increased *Prevotella* in ITB patients’ gut microbiota could further induce Th17 responses and aggravate neutrophil infiltration and pathological lesions in both lung and gut. The upregulated pro-inflammatory cytokine production may contribute to the overactivation of neutrophils and lead to impairment of mycobacterial controls within granulomas and thus exacerbate disease (Moreira-Teixeira et al., 2020). The observation of higher levels of neutrophils in the circulation of active TB patients also indicates the detrimental role of an overactivated immune response (Moideen et al., 2018). The uncontrolled replication and invasion of *M. tuberculosis* might facilitate its colonization in the gut and cause intestinal TB.

## 4 Perspectives and conclusions

The treatment of TB requires long-term multidrug treatment with a mixture of broad-spectrum and mycobacterial-specific antibiotics, especially for multidrug-resistant TB. However, it has also been reported that anti-TB medications can result in further dysbiosis of the intestinal microbiome in TB patients (Namasivayam et al., 2017; Wipperman et al., 2017; Hu et al., 2019b; Yoon et al., 2022). Intestinal microbiome disruption can also, in turn, limit the efficiency of treatment (Negi et al., 2020). A study of *M. tuberculosis* infection in mice pre-treated with isoniazid and pyrazinamide for 8 weeks also showed a higher lung bacterial burden. Besides, alleviated TNF and IL-1β production, decreased MHCII expression, and defective *M. tuberculosis* control were found in the alveolar macrophages of the mice. This phenotype can be partially reversed by fecal transplantation (Khan et al., 2019). Moreover, in our review, the current findings in TB patients also indicate a correlation between severely imbalanced gut microbiome with the development of ITB in PTB patients. Therefore, a balanced gut microbiome is crucial during *M. tuberculosis* infection. To achieve this goal, probiotics and postbiotics as potential routine supplements during TB treatment could be a one-stone-two-birds strategy.

Probiotics, such as *Bacteroides fragilis* and *Lactobacillus plantarum*, have already been considered novel probiotics in TB treatment (Liu et al., 2021; Eribo et al., 2022). *B. fragilis* has been reported to exert anti-inflammatory function by decreasing excessive IFN-γ and inducing IL-10 secretion in mice through its metabolite PSA (polysaccharide) (Johnson et al., 2015; Johnson et al., 2018). The study by Negi et al., also reported increased MHCII expression on lung dendritic cells and a lower *M. tuberculosis* burden in the lung of mice after treatment with *Lactobacillus plantarum*. Another *in vitro* study using *Lacticaseibacillus rhamnosus* PMC203 found a direct restriction in *M. tuberculosis* growth and increased killing ability in infected RAW 264.7 cells (Rahim et al., 2022).

Postbiotics, such as indole propionic acid, can inhibit *M. tuberculosis* by targeting tryptophan synthesis (Negatu et al., 2019). PBA as a derivative of probiotics (butyrate) has also been tested in clinical trials and observed to provide significant relief of symptoms (Bekele et al., 2018; Rekha et al., 2018). However, the usage and concentration of probiotics and postbiotics must be individualized in the context of the patients. For example, different concentrations of SCFAs might have distinct functions (Ashique et al., 2022). Another example is the usage of SCFAs, which might be helpful in normal TB patients, but detrimental in people with HIV co-infection (Machado et al., 2021)

As mentioned above, studies have shown that the gut microbiome alteration in general TB patients (PTB) is characterized by dysbiosis, which is defined as reduced butyrate-producing *Firmicutes* and *Prevotella* (*Bacteroidetes*), and increased lactic acid-producing *Firmicutes*, *Bacteroides*, *Parabacteroides*, and opportunistic pathogens in *Proteobacteria* and *Actinobacteria*. The most significant consequence of this alteration, given the abundance of Firmicutes and Bacteroidetes in the human gut microbiome, is the change in the composition of SCFAs, with reduced butyrate and increased acetate and propionate metabolite production. When acetate and propionate production is further decreased by the reduction of *Bacteroides*, there might be an increased susceptibility to *M. tuberculosis* infection in the gut, causing ITB. Therefore, the gut microbiome may act as the defense line in preventing ITB development. Probiotics and postbiotics could become potential supplements in TB treatment and ITB prevention.

## Author contributions

ZY and JC designed the study. ZY and XS wrote the manuscript. AW and CH made contributions to the revision. All authors contributed to the article and approved the submitted version.
